# Relationship Among Corneal Stiffness, Thickness, and Biomechanical Parameters Measured by Corvis ST, Pentacam and ORA in Keratoconus

**DOI:** 10.3389/fphys.2019.00740

**Published:** 2019-06-13

**Authors:** Yu Zhao, Yang Shen, Zhipeng Yan, Mi Tian, Jing Zhao, Xingtao Zhou

**Affiliations:** ^1^Department of Ophthalmology and Optometry, Eye and ENT Hospital, Myopia Key Laboratory of the Health Ministry, Fudan University, Shanghai, China; ^2^Shanghai Research Center of Ophthalmology and Optometry, Shanghai, China; ^3^Department of Ophthalmology, The Third Hospital of Hebei Medical University, Shijiazhuang, China

**Keywords:** kerotoconus, corneal stiffness, corneal biomechanics, Corvis ST, Pentacam

## Abstract

**Purpose:**

To assess the relationship among corneal stiffness, thickness, and biomechanical parameters in keratoconus.

**Setting:**

The EENT Hospital of Fudan University, Shanghai, China.

**Design:**

Comparative study.

**Methods:**

In this cross-sectional prospective study, 75 keratoconic eyes of 44 patients were recruited. Eyes were divided three groups according to the steepest K-readings (Kmax): mild (31 eyes; 42.1–54.5D); moderate (27 eyes, 55.0–61.6D); and severe (17 eyes, 65.2–94.5D). Thirty-one healthy subjects were recruited as the control group. All patients underwent Corvis ST, Pentacam and ORA examinations at the same time. Stiffness parameter A1 (SP-A1) and other dynamic parameters were assessed using the Corvis ST. Kmax and thinnest corneal thickness (TCT) was obtained using the Pentacam. Corneal resistance factor (CRF) and corneal hysteresis (CH) were measured using the ORA. Analysis of correlation was applied to investigate the association between variables.

**Results:**

There was a decrease in SP-A1 in different stages of keratoconus compared with controls (*P* ≤ 0.001): with increasing severity, the value of SP-A1 became smaller (*P* < 0.05). A statistically significant linear relationship was noted between SP-A1 and TCT in each subgroup of keratoconus (*P* ≤ 0.001). In all three groups, SP-A1 was found to be positively correlated with first applanation time (*P* < 0.01), while negatively correlated with deformation amplitude (*P* < 0.05). Analysis of SP-A1 with regard to CRF and CH indicated statistically positive correlation in keratoconus (*P* < 0.05).

**Conclusion:**

Significant decreases in corneal stiffness were noted in kerotoconic eyes compared with normal eyes. The stiffness parameter could be a valuable clinical tool enables biomechanically track progression with keratoconus.

**Synopsis:**

Our study found that corneal thinning and biomechanical decreasing synchronize with one another throughout the progression of the keratoconus, and SP-A1 could be a potential biomarker evaluating disease progression.

## Introduction

Keratoconus is a progressive, non-inflammatory disease in which the cornea forms a conical shape, and thins due to significant structural degeneration. It results in corneal protrusion, irregular astigmatism, and loss of visual acuity (Krachmer et al., 1984; Lawless et al., 1989). These pathological changes are considered to be the result of focal biomechanical weaknesses in the cornea (Sherwin and Brookes, 2004; Scarcelli et al., 2014, 2015) The biomechanical features of keratoconus have attracted increased research attention in the field of ophthalmology (Fontes et al., 2010; Mikielewicz et al., 2011; Galletti et al., 2012).

Previously, measurement of corneal stiffness was conducted *ex vivo* by cutting corneal strips in a specified length and placing them in a testing instrument to assess behavior. However, the *ex vivo* measurement destroys the natural state of the cornea, and is likely to be influenced by multiple factors (Elsheikh and Anderson, 2005).

The Ocular Response Analyzer (ORA; Reichert Inc, Depew, NY, United States) was introduced as the first device for evaluating corneal biomechanical parameters *in vivo*. It monitors the corneal deformation response to an air pulse, and provides biomechanics-related parameters including corneal hysteresis (CH) and corneal resistance factor (CRF). Although studies have shown that CH and CRF in kerotoconic eyes are significantly lower than in normal eyes, the specificity and sensitivity of both values are not sufficiently high to diagnosis keratoconus (Shah et al., 2007; Fontes et al., 2011).

The corneal visualization Scheimpflug tonometer (Corvis ST, Oculus Optikgeräte GmbH; Wetzlar, Germany) is an even newer instrument, which also enables the assessment of the biomechanical parameters. It uses an ultra-high speed Scheimpflug camera that enables direct visualization of corneal movement during the measurements, and provides several corneal deformation parameters. The Corvis ST has been shown to be an effective instrument aiding in the diagnosis of keratoconus (Ambrosio et al., 2006).

Recently, stiffness parameter A1 (SP-A1) was introduced as a novel parameter for corneal stiffness (Roberts et al., 2017). It is generated from initial data acquired by the Corvis ST and calculated using a particular equation. Initial studies have reported significant differences in SP-A1 between keratoconic and normal eyes. However, little study has been devoted to investigating whether corneal stiffness is altered with the progression of keratoconus. Additionally, the correlations between corneal stiffness and other corneal properties remain unclear.

In the present study, we explored the relationship among corneal stiffness, corneal thickness, and biomechanical parameters in different stages of keratoconus assessed using the Corvis ST and the ORA.

## Materials and Methods

In accordance with the tenets of the Declaration of Helsinki, the Ethics Committee of Fudan University Eye and ENT Hospital Review Board (Shanghai, China) approved the study protocol. Written informed consent was provided by all subjects before entering the study.

In this cross-sectional study, a total of 44 (75 eyes) keratoconus patients were recruited from March 2017 to June 2017 at the Department of Ophthalmology, Eye and ENT Hospital of Fudan University. The diagnosis of keratoconus was made by an experienced specialist (XZ) based on global consensus on keratoconus (Gomes et al., 2015). Because keratoconus affects both eyes in a patient unequally, all eyes that met the inclusion criteria were included. Thirty-one healthy subjects were recruited as a control group and only the right eyes were analyzed. Subjects participating in the study were 18–30 years of age. The average age of keratoconus patients (36 male, 8 female) was 22 years; the mean age of the healthy subjects (14 male, 17 female) was 25 years. Eyes with a history of other ocular diseases or any previous ocular surgery were excluded from the study.

All keratoconic eyes were divided into one of three groups according to steepest K (Kmax) readings: mild keratoconus [Kmax < 55 D (31 eyes; range, 42.1–54.5 D)], moderate keratoconus [55 D ≤ Kmax < 62 D (27 eyes; range, 55.0–61.6 D)], and severe keratoconus [Kmax ≥ 62 D (17 eyes; range, 65.2–94.5 D)]. The K-readings were obtained directly using the Pentacam HR Imaging System (Oculus Optikgeräte GmbH, Wetzlar, Germany).

Each patient underwent a comprehensive ophthalmological examination, including uncorrected distance visual acuity, manifest refraction, corrected distance visual acuity, slit-lamp examination, using the Corvis ST, Pentacam, and ORA instruments.

### Corvis ST

The Corvis ST is an imaging and tonometer that provides information regarding corneal response to an air-puff pulse. The device emits a quick, controlled air impulse to deform the cornea. A quality score is calculated after the measurement; only measurements with good quality scores were used in the statistical analyses. The following parameters are recorded during the examination: SP-A1; time, length, and corneal velocity when the cornea flattened to the first applanation (AT1, AL1, and AV1, respectively) and recovery to the second applanation (AT2, AL2, and AV2, respectively); and the time (HCT), peak to peak distance (PD), radius of central concave curvature (R), and deformation amplitude (DA) at maximum deformation.

### Pentacam

All eyes were examined using the Pentacam HR imaging system (Oculus Optikgeräte GmbH, Wetzlar, Germany). Patients were asked to fixate their eye on a target light. After attaining alignment, the device captures 25, 360°images automatically in 2 s. To avoid miscalculations of poor imaging quality, the quality of the measurement results are displayed in a specification window; only results with “OK” statements were accepted. The examination was duplicated if the comment was marked in yellow or red. Kmax, thinnest corneal thickness (TCT), and posterior corneal elevation at the thinnest point (PCE) data were acquired using the Pentacam instrument.

### ORA

The ocular response analyzer generates a precise stream of air to indent the cornea. During the process, an infrared detector measured the number of photons reflected from the corneal center. Dynamical parameters are produced based on two distinct peaks: pressure 1 in the inward direction and pressure 2 in the outward direction. CH is defined as the difference between these two pressures, reflecting the ability of the corneal to absorb energy. The CRF, as calculated by the formula, represents the entire corneal resistance to the deformation. Measurements were repeated until the signal score was >4.0, the values of CH and CRF were recorded.

### Statistical Analysis

Descriptive results are presented as mean and standard deviation. The Kolmogorov-Smirnow normality test and Levene test for equal variances were executed for all data. Differences between keratoconic and normal eyes were compared using the independent *t*-test or the Wilcoxon rank-sum test. Bivariate normal analysis was executed before the correlation test. The Pearson or Spearman correlation test was applied subsequently to determine the association between variables. Stepwise multiple linear regression model analysis was performed to predict theoretical SP-A1 values. Statistical analyses were performed using SPSS version 22.0 (SPSS Inc., Chicago, IL, United States). *P* < 0.05 was considered to indicate a statistically significant difference.

## Results

**FIGURE 1:**
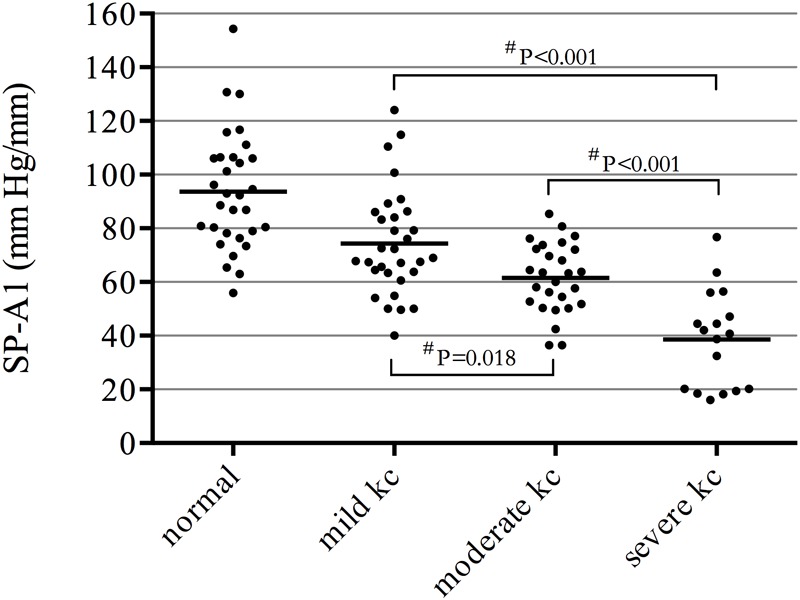
Significant decrease in stiffness parameter A1 (SP-A1) in all three groups of keratoconus patients compared with controls (*P* ≤ 0.001); decreasing in various levels of severity of keratoconus presented significant statistical difference between each two groups. ^#^ANOVA with the Bonferroni correction.

There was a significant decrease in SP-A1 in all three groups of keratoconus patients compared with controls (*P* < 0.001). The more severe the disease, the smaller the value was. Decreasing in various levels of severity of keratoconus presented significant statistical difference between each two groups (Figure 1). Additionally, SP-A1 and most of the measured parameters demonstrated a statistical difference in the mild keratoconus and control groups (Table 1).

**Table 1:** Relationship between all measured parameters in normal compared with keratoconus and mild keratoconus.

Parameter	Average ± SD						
	Normal eyes	Keratoconic eyes				Normal vs. KC, P	Normal vs. Mild, P
		All	Mild	Moderate	Severe		
SP-A1	93.68 ± 21.93	61.62 ± 21.87	74.85 ± 19.35	60.43 ± 12.65	38.87 ± 17.66	0.000^#b^	0.001^#b^
AT1	7.23 ± 0.28	6.92 ± 0.40	6.99 ± 0.36	6.98 ± 0.32	6.70 ± 0.48	0.000^*b^	0.001^#b^
AL1	2.31 ± 0.35	1.91 ± 0.38	2.00 ± 0.41	1.93 ± 0.32	1.70 ± 0.31	0.000^*b^	0.003^#b^
AV1	0.14 ± 0.02	0.18 ± 0.12	0.19 ± 0.18	0.16 ± 0.02	0.20 ± 0.04	0.000^*b^	0.020^*a^
AT2	21.56 ± 0.51	21.66 ± 0.58	21.66 ± 0.46	21.49 ± 0.61	21.99 ± 0.53	0.398^#^	0.406^#^
AL2	1.83 ± 0.47	1.47 ± 0.43	1.57 ± 0.38	1.46 ± 0.48	1.42 ± 0.41	0.000^#b^	0.016^#a^
AV2	-0.28 ± 0.05	-0.33 ± 0.32	-0.36 ± 0.48	-0.27 ± 0.08	-0.37 ± 0.08	0.188^*^	0.547^*^
HCT	16.80 ± 0.53	16.54 ± 0.66	16.66 ± 0.58	16.59 ± 0.51	16.32 ± 0.94	0.054^#^	0.205^#^
PD	4.98 ± 0.38	4.99 ± 0.33	5.11 ± 0.30	4.92 ± 0.24	4.84 ± 0.40	0.883^#^	0.061^#^
R	6.67 ± 0.90	5.02 ± 1.10	5.89 ± 0.68	4.80 ± 0.62	3.94 ± 1.19	0.000^#b^	0.000^#b^
DA	1.04 ± 0.10	1.33 ± 1.26	1.12 ± 0.10	1.49 ± 1.95	1.37 ± 0.22	0.000^*b^	0.003^#b^
Kmax	44.42 ± 1.59	57.62 ± 10.76	48.39 ± 3.75	58.23 ± 1.97	74.13 ± 8.44	0.000^*b^	0.000^*b^
TCT	540.58 ± 21.93	453.64 ± 81.53	488.70 ± 27.86	469.13 ± 34.73	364.11 ± 115.99	0.000^*b^	0.000^*b^
PCE	6.06 ± 3.18	60.61 ± 60.54	26.82 ± 14.71	56.97 ± 14.37	126.69 ± 92.21	0.000^*b^	0.000^*b^
CH	10.91 ± 1.56	9.01 ± 1.78	9.3 ± 1.78	9.23 ± 1.77	8.18 ± 1.43	0.000^#b^	0.000^#b^
CRF	10.95 ± 2.00	8.38 ± 2.21	12.13 ± 1.993	8.57 ± 1.95	6.79 ± 2.27	0.000^#b^	0.000^#b^

*^#^Independent *t*-test. ^∗^Wilcoxon rank-sum test. ^*a*^Difference is significant at the 0.05 level. ^*b*^Difference is significant at the 0.01 level.*

**FIGURE 2:**
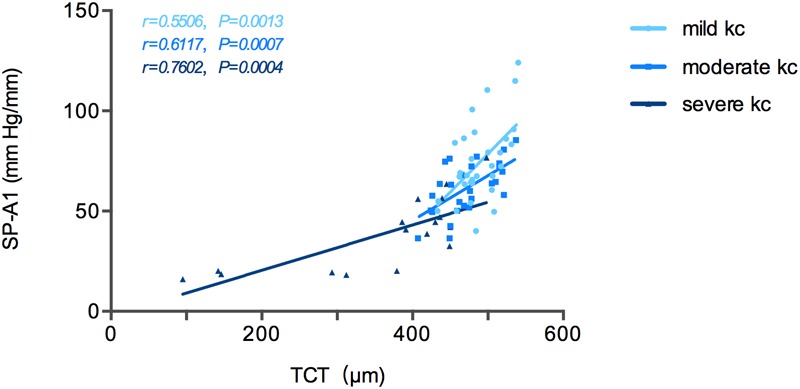
Stiffness parameter A1 (SP-A1) was significantly and positively correlated with thinnest corneal thickness (TCT) in keratoconus group.

Stiffness parameter A1 was significantly and positively correlated with Kmax in the mild keratoconus group (*r* = -0.533, *P* = 0.002), and with TCT in all three groups [mild (keratoconus) group: *r* = 0.551, *P* = 0.001; moderate group: *r* = 0.612, *P* = 0.001; severe group: *r* = 0.760, *P* < 0.001; Figure 2]. In severe keratoconus group, SP-A1 was found negatively correlated with PCE (*r* = -0.554, *P* = 0.021).

**FIGURE 3:**
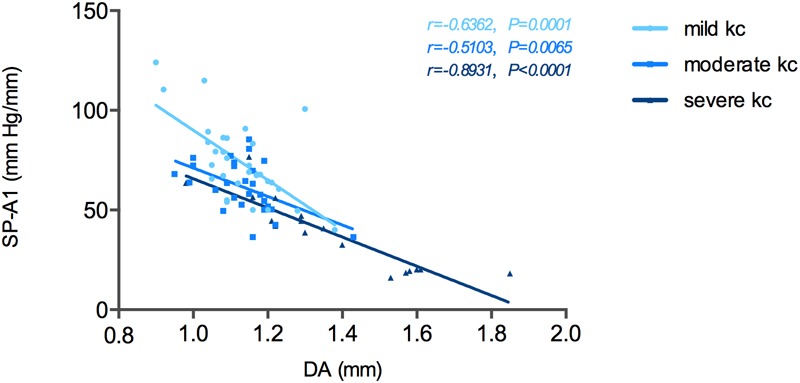
Stiffness parameter A1 (SP-A1) was significantly and negatively correlated with deformation amplitude (DA) in keratoconus group.

The relationship between SP-A1 and the original Corvis ST-acquired values was also analyzed. For all different stages of keratoconus eyes, a significant positive relationship was noted between SP-A1 and AT1 (*P* = 0.003). Additionally, there was negative statistical correlation between SP-A1 and DA [mild (keratoconus) group: *r* = -0.636, *P* < 0.001; moderate group: *r* = -0.468, *P* = 0.012; severe group: *r* = -0.909, *P* < 0.001; Figure 3]. No statistically significant relationship was demonstrated in tomography features and original Corvis ST values in all keratoconus groups.

**FIGURE 4:**
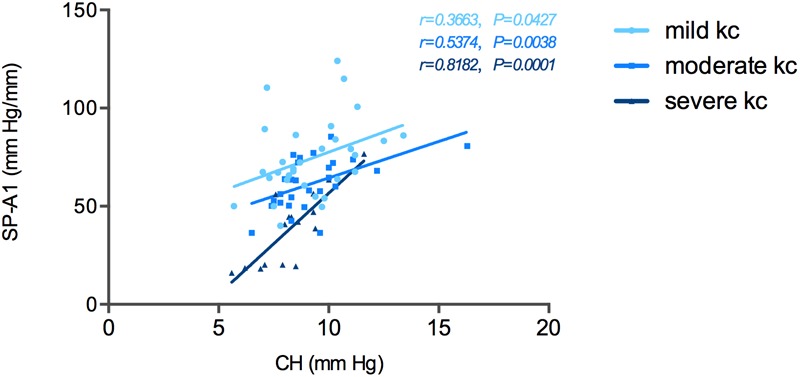
Stiffness parameter A1 (SP-A1) was significantly and positively correlated with corneal hysteresis (CH) in keratoconus group.

**FIGURE 5:**
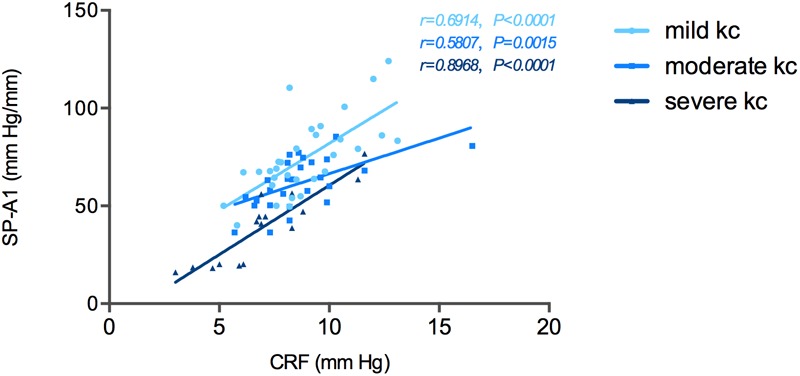
Stiffness parameter A1 (SP-A1) was significantly and positively correlated with corneal resistance factor (CRF) in keratoconus group.

Correlation tests of SP-A1 and CH demonstrated a significant positive relationship between the two in keratoconic eyes [mild (keratoconus) group: *r* = 0.366, *P* = 0.043; moderate group: *r* = 0.537, *P* = 0.003; severe group: *r* = 0.818, *P* < 0.001; Figure 4]. Similar results were found between SP-A1 and CRF [mild (keratoconus) group: *r* = 0.666, *P* < 0.001; moderate group: *r* = 0.581, *P* = 0.001; severe group: *r* = 0.897, *P* < 0.001; Figure 5]. When analyzing the relationship between tomography features and CH and CRF, only TCT correlated positively with the two parameters in various degrees of keratoconus (*P* < 0.05).

Statistically significant results in correlation tests for various degrees of keratoconus and normal controls are summarized in Tables 2–5. The results of multiple linear regression model analysis were represented in Table 6.

**Table 2:** Significant statistical correlation between parameters in mild kerotoconus.

		SPA1	AT1	AL1	AV1	AT2	AV2	HCT	PD	*R*	DA	Kmax	TCT	PCE	CH	CRF
SPA1	r		0.589		-0.618^#^	-0.663			-0.38	0.409	-0.636	-0.533	0.551		0.366	0.666
	P		0.000^#b^		0.000^*b^	0.000^#b^			0.035^#a^	0.022^#a^	0.000^#b^	0.002^#b^	0.001^#b^		0.043^#a^	0.000^*b^
AT1	r	0.589				-0.399			-0.395		-0.481					
	P	0.000^#b^				0.026^#a^			0.028^#a^		0.006^#b^					
AL1	r				-0.449											
	P				0.011^*a^											
AV1	r	-0.618		-0.449						-0.441	0.377	0.375	-0.505			-0.442
	P	0.000^*b^		0.011^*a^						0.013^*a^	0.036^*a^	0.037^*a^	0.004^*b^			0.013^*a^
AT2	r	-0.663	-0.399					0.493	0.651		0.634					-0.449
	P	0.000^#b^	0.026^#a^					0.005^*b^	0.000^#b^		0.000^#b^					0.011^*a^
AL2	r															
	P															
AV2	r								-0.493		-0.681					
	P								0.005^*b^		0.000^*b^					
HCT	r					0.493										
	P					0.005^*b^										
PD	r	-0.38	-0.395			0.651	-0.493			0.387	0.805					
	P	0.035^#a^	0.028^#a^			0.000^#b^	0.005^#b^			0.031^#a^	0.000^#b^					
R	r	0.409			-0.441				0.387			-0.531	0.416	-0.441		
	P	0.022^#a^			0.013^*a^				0.031^#a^			0.002^#b^	0.020^#a^	0.013^#^		
DA	r	-0.636	-0.481		0.377	0.634	-0.681		0.805							-0.392
	P	0.000^#b^	0.006^#b^		0.036^*a^	0.000^#b^	0.000^#b^		0.000^#b^							0.029^*a^
Kmax	r	-0.533			0.375					-0.531			-0.694	0.722	-0.538	-0.513
	P	0.002^#b^			0.037^*a^					0.002^#b^			0.000^#b^	0.000^#^	0.002^#b^	0.003^*b^
TCT	r	0.551			-0.505					0.416		-0.694		-0.545	0.505	0.453
	P	0.001^#b^			0.004^*b^					0.020^#a^		0.000^#b^		0.002^#^	0.004^#b^	0.011^*a^
PCE	r									-0.441		0.722	-0.545		-0.359	
	P									0.013^#a^		0.000^#b^	0.002^#b^		0.047^#a^	
CH	r	0.366										-0.538	0.505	-0.359		0.824
	P	0.043^#a^										0.002^#b^	0.004^#b^	0.047^#^		0.000^*b^
CRF	r	0.666			-0.442	-0.449					-0.392	-0.513	0.453		0.824	
	P	0.000^*b^			0.013^*a^	0.011^*a^					0.029^*a^	0.003^*b^	0.011^*a^		0.000^*b^	

*^#^Pearson correlation test. ^∗^Spearman correlation test. ^*a*^Difference is significant at the 0.05 level. ^*b*^Difference is significant at the 0.01 level.*

**Table 3:** Significant statistical correlation between parameters in moderate kerotoconus.

		SPA1	AT1	AL1	AV1	AT2	AL2	PD	*R*	DA	TCT	CH	CRF
SPA1	r		0.702					-0.395		-0.468	0.612	0.537	0.581
	P		0.000^#b^					0.038^#a^		0.012^*a^	0.001^#b^	0.003^#b^	0.001^#b^
AT1	r	0.702						-0.752		-0.683			0.408
	P	0.000^#b^						0.000^#b^		0.000^*b^			0.031^#a^
AL1	r				-0.736		0.415						
	P				0.000^*b^		0.028^*a^						
AV1	r			-0.736						0.471			
	P			0.000^*b^						0.011^*a^			
AT2	r						0.401						
	P						0.034^#a^						
AL2	r			0.415		0.401							
	P			0.028^*a^		0.034^#a^							
AV2	r												
	P												
HCT	r												
	P												
PD	r	-0.395	-0.752						0.374	0.525			
	P	0.038^#a^	0.000^#b^						0.050^#a^	0.004^*b^			
R	r							0.374			0.502		
	P							0.050^#a^			0.006^#b^		
DA	r	-0.468	-0.683		0.471			0.525					
	P	0.012^*a^	0.000^*b^		0.011^*a^			0.004^*b^					
Kmax	r												
	P												
TCT	r	0.612							0.502			0.427	0.421
	P	0.001^#b^							0.006^#b^			0.023^#a^	0.026^#a^
PCE	r												
	P												
CH	r	0.537									0.427		0.889
	P	0.003^#b^									0.023^#a^		0.000^#b^
CRF	r	0.581	0.408								0.421	0.889	
	P	0.001^#b^	0.031^#a^								0.026^#a^	0.000^#b^	

*^#^Pearson correlation test. ^∗^Spearman correlation test. ^*a*^Difference is significant at the 0.05 level. ^*b*^Difference is significant at the 0.01 level.*

**Table 4:** Significant statistical correlation between parameters in severe kerotoconus.

		SPA1	AT1	AL1	AV1	AT2	AL2	AV2	PD	*R*	DA	Kmax	TCT	PCE	CH	CRF
SPA1	r		0.674		-0.509			0.83	-0.546		-0.908		0.760	-0.554	0.818	0.897
	P		0.003^#b^		0.037^#a^			0.000^*b^	0.023^#a^		0.000^*b^		0.000^#b^	0.021^#a^	0.000^#b^	0.000^#b^
AT1	r	0.674				-0.729		0.538			-0.736		0.504		0.516	0.651
	P	0.003^#b^				0.001^#b^		0.026^*a^			0.001^*b^		0.039^#a^		0.041^#a^	0.006^#b^
AL1	r				-0.726		0.537									
	P				0.001^#b^		0.026^#a^									
AV1	r	-0.509		-0.726											-0.500	-0.573
	P	0.037^#a^		0.001^#b^											0.041^#a^	0.020^#a^
AT2	r		-0.729								0.563					
	P		0.001^#b^								0.019^*a^					
AL2	r			0.537						0.505						
	P			0.026^#a^						0.039^*a^						
AV2	r	0.83	0.538								-0.905		0.537		0.636	0.729
	P	0.000^*b^	0.026^*a^								0.000^*b^		0.026^#a^		0.008^#b^	0.001^#b^
HCT	r															
	P															
PD	r	-0.546													-0.603	-0.611
	P	0.023^#a^													0.013^#a^	0.012^#a^
R	r						0.505					-0.545				
	P						0.039^*a^					0.024^#a^				
DA	r	-0.908	-0.736			0.563		-0.905					-0.678		-0.697	-0.791
	P	0.000^*b^	0.001^*b^			0.019^*a^		0.000^*b^					0.003^#b^		0.003^#b^	0.000^#b^
Kmax	r									-0.545						
	P									0.024^#a^						
TCT	r	0.760	0.504					0.537			-0.678			-0.727	0.828	0.825
	P	0.000^#b^	0.039^#a^					0.026^#a^			0.003^#b^			0.001^#b^	0.000^#b^	0.000^#b^
PCE	r	-0.554											-0.727		-0.646	-0.625
	P	0.021^#a^											0.001^#b^		0.007^#b^	0.010^#b^
CH	r	0.818	0.516					0.636	-0.603		-0.697		0.828	-0.646		0.956
	P	0.000^#b^	0.041^#a^					0.008^#b^	0.013^#a^		0.003^#b^		0.000^#b^	0.007^#b^		0.000^#b^
CRF	r	0.897	0.651		-0.573			0.729	-0.611		-0.791		0.825	-0.625	0.956	
	P	0.000^#b^	0.006^#b^		0.020^#a^			0.001^#b^	0.012^#a^		0.000^#b^		0.000^#b^	0.010^#b^	0.000^#b^	

*^#^Pearson correlation test. ^∗^Spearman correlation test. ^*a*^Difference is significant at the 0.05 level. ^*b*^Difference is significant at the.01 level.*

**Table 5:** Significant statistical correlation between parameters in normal controls.

		SPA1	AT1	AV1	AT2	AL2	AV2	HCT	PD	*R*	DA	Kmax	TCT	CH	CRF
SPA1	r		0.479	-0.831	-0.667		0.770		-0.543		-0.740				0.535
	P		0.006^#b^	0.000^#b^	0.000^#b^		0.000^*b^		0.002^*b^		0.000^*b^				0.002^#b^
AT1	r	0.479		-0.718	-0.556		0.421		-0.776		-0.684				0.584
	P	0.006^#b^		0.000^#b^	0.001^#b^		0.018^#a^		0.000^#b^		0.000^#b^				0.001^#b^
AL1	r														
	P														
AV1	r	-0.831	-0.718		0.724		-0.742		0.581		0.750				-0.555
	P	0.000^#b^	0.000^#b^		0.000^#b^		0.000^#b^		0.001^#b^		0.000^#b^				0.001^#b^
AT2	r	-0.667	-0.556	0.724			-0.409		0.657	0.360	0.627				
	P	0.000^#b^	0.001^#b^	0.000^#b^			0.022^#a^		0.000^#b^	0.047^#a^	0.000^#b^				
AL2	r										-0.495				
	P										0.005^#b^				
AV2	r	0.770	0.421	-0.742	-0.409						-0.789				0.492
	P	0.000^*b^	0.018^#a^	0.000^#b^	0.022^#a^						0.000^#b^				0.005^#b^
HCT	r												0.440	0.382	
	P												0.013^#a^	0.034^#a^	
PD	r	-0.543	-0.776	0.581	0.657					0.427	0.705	-0.456			-0.472
	P	0.002^*b^	0.000^#b^	0.001^#b^	0.000^#b^					0.017^#a^	0.000^#b^	0.010^#b^			0.007^#b^
R	r				0.360				0.427						
	P				0.047^#a^				0.017^#a^						
DA	r	-0.740	-0.684	0.750	0.627	-0.495	-0.789		0.705			-0.370			-0.529
	P	0.000^*b^	0.000^#b^	0.000^#b^	0.000^#b^	0.005^#b^	0.000^#b^		0.000^#b^			0.041^#a^			0.002^#b^
Kmax	r								-0.456		-0.370				
	P								0.010^#b^		0.041^#a^				
TCT	r							0.440						0.367	
	P							0.013^#a^						0.042^#a^	
PCE	r														
	P														
CH	r							0.382					0.367		0.823
	P							0.034^#a^					0.042^#a^		0.000^#b^
CRF	r	0.535	0.584	-0.555			0.492		-0.472		-0.529			0.823	
	P	0.002^#b^	0.001^#b^	0.001^#b^			0.005^#b^		0.007^#b^		0.002^#b^			0.000^#b^	

*^#^Pearson correlation test. ^∗^Spearman correlation test. ^*a*^Difference is significant at the 0.05 level. ^*b*^Difference is significant at the 0.01 level.*

**Table 6:** The stepwise multiple linear regression model analysis for predicting theoretical SP-A1 in keratoconus.

Main predictors	*B*	*SE*	β	*t*	Sig.	Adjusted *R*^2^	*F*	Sig.
Constant	45.8	17.440		2.626	0.011	0.743	43.224	<0.001
Kmax	-0.707	0.163	-0.344	-4.328	<0.001			
TCT	0.073	0.030	0.274	2.417	0.018			
CH	-5.206	1.538	-0.425	-3.385	0.001			
CRF	8.22	1.375	0.837	5.978	<0.001			

*The regression equation is SPA1 = 45.8- 0.707Kmax+0.073TCT-5.206CH+8.222CRF. B, unstandarized coefficients; SE, standard error of unstandardized coefficients; β, standardized coefficients (beta); t, Unstandardized coefficients/standard error; Sig, significance.*

## Discussion

Published studies have suggested that a reduction in biomechanical properties plays an important role in the generation and progression of keratoconus (Meek et al., 2005; Morishige et al., 2007). However, until recently, assessment for relating corneal properties to the severity of keratoconus has been restricted by the lack of measuring instruments. SP-A1, the first corneal stiffness value recorded *in vivo* by the Corvis ST, was developed by using displacement of the apex from the undeformed state to first applanation in the deformation process. It takes into account confounding factors such as intraocular pressure and whole eye motion. The value is anticipated to be a useful indicator of corneal resistance to deformation, and shows promise in the evaluation of keratoconus (Vinciguerra et al., 2016; Roberts et al., 2017). By studying the correlation between SP-A1 and other corneal parameters in various degrees of keratoconus, we may gain further insight into the relationship between biomechanical and morphological features of the disease.

In the current study, a decrease in SP-A1 of keratoconus compared with controls was demonstrated; significant statistical difference between various levels of severity of keratoconus was presented as well. Additionally, a statistically positive correlation was revealed between SP-A1 and TCT in all keratoconus groups, and the relative index increased with the severity of keratoconus. Theoretical models of keratoconus etiology have proposed that loss of structural integrity initiates the reduction in biomechanical properties, which in turn lead to focal weakening in the cornea of keratoconus patients (Sinha Roy and Dupps, 2011; Roberts and Dupps, 2014). Subsequently, the focal area will strain to a greater extent than the surrounding normal area under the same intraocular pressure. This results in thinning of the cornea, further decreasing its biomechanical properties, and further thinning. The outcomes of the present study provide support for this hypothesis, and these two features synchronize with one another throughout the progression of the disease. Describing reduced corneal biomechanical stability occurs prior to alteration of corneal shape, SP-A1 could be a potential biomarker evaluating progression of keratoconus.

It is worth noting that no statistical correlation was demonstrated between SP-A1 and TCT in the normal controls. The low sample size in the normal group should be taken into consideration firstly, which could lead to an insignificant statistical result. Apart from this, there may be other explanations. It has been established that the corneal stroma is primarily composed of collagen lamellae and constitutes approximately 90% of the total thickness of the cornea (Meek et al., 2005). The results suggest that although the majority of the stiffness in the normal cornea arises from layers of collagen lamellae, factors, including extracellular matrix and intensity of collagen cross-links, and may also play an indispensable role in corneal biomechanical support (Andreassen et al., 1980). For keratoconous, breaks in Bowman’s layer, atypical organization of collagen fibrils, reduced cross-links, and other pathological changes are likely responsible for corneal weakness and, thus, may increase the contribution of corneal thickness to stiffness (Matthews et al., 2007).

A negative correlation between SP-A1 and DA was identified in all keratoconus groups, and keratoconic eyes exhibited greater DA than normal eyes. The present study yielded results similar to those reported in several previous investigations. Ali et al. (2014) assessed dynamic parameters in 45 keratoconic eyes and reported a higher DA and a lower AT1 in keratoconic eyes than in control eyes. When controlled for intraocular pressure and corneal thickness, DA was also statistically greater in keratoconic eyes than controls. Tian et al. (2014) also compared original data acquired using the Corvis ST in keratoconus vs. normal patients, and found that DA demonstrated a stronger sensitivity for detection of keratoconus than other original data. These results are consistent with those from the present study: the weaker the cornea, the more prone to deformation and, therefore, greater deformation results.

Previously, direct comparisons between the Corvis ST and ORA were not possible because the two systems were based on different principles (i.e., Corvis ST emits a constant metered air pulse, while ORA has a variable maximum air pressure) (Vellara and Patel, 2015). By analyzing the new corneal stiffness parameter, we found that SP-A1 was positively correlated with CRF in keratoconus, as well as in normal controls. In a previous report, Dupps (2007) explained that CRF comprises both elastic resistance and viscous damping, which reflects resistance to deformation of the entire cornea. Similarly, SP-A1 reflects corneal stiffness and is closely related to corneal properties. The two parameters could share similarities in evaluating corneal conditions and predicting ectasia progression. This was the first study to demonstrate a correlation between data acquired using the Corvis ST and ORA systems; research aimed at validating the outcomes of the present investigation is warranted.

There were limitations to this study, the first of which was the relatively small sample size, which may have limited the interpretation of the statistical analysis. Second, because keratoconic eyes were divided into different stages according to diopter, corneal thickness could be an influencing factor in analyzing correlations between SP-A1 and other parameters.

In conclusion, significant decreases in corneal stiffness were noted in kerotoconic eyes compared with normal eyes. Attempts to improve the diagnostic efficiency of the stiffness parameter for mild kerotoconus should be pursued in future studies.

## What Was Known

• A reduction in biomechanical properties plays an important role in the generation and progression of keratoconus.

## What This Paper Adds

• Decreases in corneal stiffness were noted in kerotoconic eyes compared with normal eyes; significant statistical difference between various levels of severity of keratoconus was presented as well.
• Corneal thinning and biomechanical decreasing synchronize with one another throughout the progression of the keratoconus and SP-A1 could be a potential biomarker evaluating disease progression.

## Ethics Statement

This study was carried out in accordance with the recommendations of tenets of the Declaration of Helsinki with written informed consent from all subjects. All subjects gave written informed consent in accordance with the Declaration of Helsinki. The protocol was approved by the Ethics Committee of Fudan University Eye and ENT Hospital Review Board (Shanghai, China).

## Author Contributions

ZY, YS, and YZ performed the initial clinical database search. MT and JZ completed the statistical analysis. ZY produced the first draft of the manuscript and figures. All authors contributed to the study revision and edited the manuscript. XZ supervised the study and contributed to the final approval of the version sent for approval.

## Conflict of Interest Statement

The authors declare that the research was conducted in the absence of any commercial or financial relationships that could be construed as a potential conflict of interest.

## References

[B1] Ali N. Q. Patel D. V. McGhee C. N. (2014). Biomechanical responses of healthy and keratoconic corneas measured using a noncontact scheimpflug-based tonometer. *Invest. Ophthalmol. Vis. Sci.* 55 3651–3659. 10.1167/iovs.13-13715 24833745

[B2] Ambrosio R. Jr. Alonso R. S. Luz A. Coca Velarde L. G. (2006). Corneal-thickness spatial profile and corneal-volume distribution: tomographic indices to detect keratoconus. *J. Cataract. Refract. Surg.* 32 1851–1859. 10.1016/j.jcrs.2006.06.025 17081868

[B3] Andreassen T. T. Simonsen A. H. Oxlund H. (1980). Biomechanical properties of keratoconus and normal corneas. *Exp. Eye Res.* 31 435–441. 10.1016/s0014-4835(80)80027-3 7449878

[B4] Dupps W. J. Jr. (2007). Hysteresis: new mechanospeak for the ophthalmologist. *J. Cataract. Refract. Surg.* 33 1499–1501. 10.1016/j.jcrs.2007.07.008 17720051

[B5] Elsheikh A. Anderson K. (2005). Comparative study of corneal strip extensometry and inflation tests. *J. R. Soc. Interface* 2 177–185. 10.1098/rsif.2005.0034 16849178 PMC1629069

[B6] Fontes B. M. Ambrosio R. Jr. Jardim D. Velarde G. C. Nose W. (2010). Corneal biomechanical metrics and anterior segment parameters in mild keratoconus. *Ophthalmology* 117 673–679. 10.1016/j.ophtha.2009.09.023 20138369

[B7] Fontes B. M. Ambrosio R. Jr. Velarde G. C. Nose W. (2011). Ocular response analyzer measurements in keratoconus with normal central corneal thickness compared with matched normal control eyes. *J. Refract. Surg.* 27 209–215. 10.3928/1081597X-20100415-02 20481414

[B8] Galletti J. G. Pfortner T. Bonthoux F. F. (2012). Improved keratoconus detection by ocular response analyzer testing after consideration of corneal thickness as a confounding factor. *J. Refract. Surg.* 28 202–208. 10.3928/1081597X-20120103-03 22230059

[B9] Gomes J. A. Tan D. Rapuano C. J. Belin M. W. Ambrosio R. Jr. Guell J. L. (2015). Global consensus on keratoconus and ectatic diseases. *Cornea* 34 359–369. 10.1097/ICO.0000000000000408 25738235

[B10] Krachmer J. H. Feder R. S. Belin M. W. (1984). Keratoconus and related noninflammatory corneal thinning disorders. *Surv. Ophthalmol.* 28 293–322. 10.1016/0039-6257(84)90094-8 6230745

[B11] Lawless M. Coster D. J. Phillips A. J. Loane M. (1989). Keratoconus: diagnosis and management. *Aust. N. Z. J. Ophthalmol.* 17 33–60. 2527524 10.1111/j.1442-9071.1989.tb00487.x

[B12] Matthews F. J. Cook S. D. Majid M. A. Dick A. D. Smith V. A. (2007). Changes in the balance of the tissue inhibitor of matrix metalloproteinases (TIMPs)-1 and -3 may promote keratocyte apoptosis in keratoconus. *Exp. Eye Res.* 84 1125–1134. 10.1016/j.exer.2007.02.013 17449031

[B13] Meek K. M. Tuft S. J. Huang Y. Gill P. S. Hayes S. Newton R. H. (2005). Changes in collagen orientation and distribution in keratoconus corneas. *Invest. Ophthalmol. Vis. Sci.* 46 1948–1956. 15914608 10.1167/iovs.04-1253

[B14] Mikielewicz M. Kotliar K. Barraquer R. I. Michael R. (2011). Air-pulse corneal applanation signal curve parameters for the characterisation of keratoconus. *Br. J. Ophthalmol.* 95 793–798. 10.1136/bjo.2010.188300 21310802

[B15] Morishige N. Wahlert A. J. Kenney M. C. Brown D. J. Kawamoto K. Chikama T. (2007). Second-harmonic imaging microscopy of normal human and keratoconus cornea. *Invest. Ophthalmol. Vis. Sci.* 48 1087–1094. 17325150 10.1167/iovs.06-1177 PMC1894888

[B16] Roberts C. J. Dupps W. J. Jr. (2014). Biomechanics of corneal ectasia and biomechanical treatments. *J. Cataract Refract. Surg.* 40 991–998. 10.1016/j.jcrs.2014.04.013 24774009 PMC4850839

[B17] Roberts C. J. Mahmoud A. M. Bons J. P. Hossain A. Elsheikh A. Vinciguerra R. (2017). Introduction of two novel stiffness parameters and interpretation of air puff-induced biomechanical deformation parameters with a dynamic scheimpflug analyzer. *J. Refract. Surg.* 33 266–273. 10.3928/1081597X-20161221-03 28407167

[B18] Scarcelli G. Besner S. Pineda R. Kalout P. Yun S. H. (2015). In vivo biomechanical mapping of normal and keratoconus corneas. *JAMA Ophthalmol.* 133 480–482. 25611213 10.1001/jamaophthalmol.2014.5641 PMC4698984

[B19] Scarcelli G. Besner S. Pineda R. Yun S. H. (2014). Biomechanical characterization of keratoconus corneas ex vivo with Brillouin microscopy. *Invest. Ophthalmol. Vis. Sci.* 55 4490–4495. 10.1167/iovs.14-14450 24938517 PMC4109405

[B20] Shah S. Laiquzzaman M. Bhojwani R. Mantry S. Cunliffe I. (2007). Assessment of the biomechanical properties of the cornea with the ocular response analyzer in normal and keratoconic eyes. *Invest. Ophthalmol. Vis. Sci.* 48 3026–3031. 17591868 10.1167/iovs.04-0694

[B21] Sherwin T. Brookes N. H. (2004). Morphological changes in keratoconus: pathology or pathogenesis. *Clin. Exp. Ophthalmol.* 32 211–217. 10.1111/j.1442-9071.2004.00805.x 15068441

[B22] Sinha Roy A. Dupps W. J. Jr. (2011). Patient-specific computational modeling of keratoconus progression and differential responses to collagen cross-linking. *Invest. Ophthalmol. Vis. Sci.* 52 9174–9187. 10.1167/iovs.11-7395 22039252 PMC3253542

[B23] Tian L. Huang Y. F. Wang L. Q. Bai H. Wang Q. Jiang J. J. (2014). Corneal biomechanical assessment using corneal visualization scheimpflug technology in keratoconic and normal eyes. *J. Ophthalmol.* 2014:147516. 10.1155/2014/147516 24800059 PMC3988970

[B24] Vellara H. R. Patel D. V. (2015). Biomechanical properties of the keratoconic cornea: a review. *Clin. Exp. Optom.* 98 31–38. 10.1111/cxo.12211 25545947

[B25] Vinciguerra R. Ambrosio R. Jr. Elsheikh A. Roberts C. J. Lopes B. Morenghi E. (2016). Detection of keratoconus with a new biomechanical index. *J. Refract. Surg.* 32 803–810. 10.3928/1081597X-20160629-01 27930790

